# Management of hereditary angioedema: 2010 Canadian approach

**DOI:** 10.1186/1710-1492-6-20

**Published:** 2010-07-28

**Authors:** Tom Bowen, John Brosz, Kristylea Brosz, Jacques Hebert, Bruce Ritchie

**Affiliations:** 1 Canadian Hereditary Angioedema Network (CHAEN)/Réseau Canadien d'Angioédème Héréditaire (RCAH), 705 South Tower, 3031 Hospital Dr. NW Calgary, Alberta, Canada; 2 Departments of Medicine and Paediatrics, University of Calgary, Calgary, Alberta, Canada; 3 Patient Advisory Committee, CHAEN/RCAH. 705 South Tower, 3031 Hospital Dr. NW, Calgary, Alberta, Canada; 4 Department of Medicine, Laval University, Quebec City, Quebec, Canada; 5 Departments of Medicine and Medical Oncology, University of Alberta, Edmonton, Alberta, Canada

## Abstract

C1-inhibitor (C1-INH) deficiency is a rare blood disorder resulting in angioedema attacks that are debilitating and may be life-threatening. Prophylaxis and therapy of events has changed since our first Canadian Consensus Conference on the diagnosis, therapy and management of HAE. We have formed the **Canadian Hereditary Angioedema Network (CHAEN)/Réseau Canadien d'Angioédème Héréditaire (RCAH) **- http://www.haecanada.com to advance care of patients with this disorder in Canada. We here present a review of management of HAE in Canada.

## Introduction

C1 inhibitor (C1-INH) deficiency presents in congenital (hereditary angioedema, HAE) or acquired forms. There are three variants of hereditary angioedema (HAE): HAE-C1-INH Type I with low C1-INH protein and function (85% of cases; autosomal dominant); HAE-C1-INH Type II with normal protein but low function (15% of cases; autosomal dominant); and HAE Type III hereditary angioedema with normal C1 inhibitor protein and function (estrogen-dependent inherited form found mostly in females; some with defects in coagulation factor XII, HAE-FXII; others of unknown defect, HAE-Unknown) [1,2]. Acquired angioedema (AAE) is most frequently associated with lymphoproliferative and autoimmune disorders and with some medications including ACE inhibitors and plasmin activators [3,4]. Patients with HAE may experience recurrent soft tissue swellings, intestinal swellings, and abdominal pains, and may have life-threatening swellings of the airway. The incidence of HAE is estimated at 1:10,000 to 1:50,000. Risk of dying from airway obstruction is not clear but deaths from this complication if left untreated are not uncommon [1,5].

To learn from international experience in HAE, the first C1-INH Deficiency Workshop was convened by the Hungarian HAE Center in May 1999 and they have organized ongoing workshops every two years. The 6^th ^International C1-INH Deficiency Workshop was held in May 2009 in Budapest http://www.haenet.hu/new/index2.html.

In Canada, Jeanne Burnham organized the HAE patient organization and Scientific Advisory Committee interested in advancing the standard of care for HAE in Canada and from this grew the Canadian Hereditary Angioedema Society (CHAES)/Société d'angioédème héréditaire du Canada (SAHC) established in 2001. This group recently disbanded and evolved into an informal network of HAE physicians with patient advisory input, the **Canadian Hereditary Angioedema Network (CHAEN)/Réseau Canadien d'Angioédème Héréditaire (RCAH) **- http://www.haecanada.com. The first meeting of CHAEN/RCAH took place together with the Canadian Society of Allergy and Clinical Immunology/La Société canadienne d'allergie et d'immunologie clinique (CSACI/SCAIC) in Edmonton, Alberta, Canada September 2007 and its second meeting in Toronto, Ontario, Canada May 16^th^, 2010 along with the Canadian HAE Consensus 2010 Conference http://www.haecanada.com/m.php?p=ehome. In 2002, we proposed to coordinate therapy for HAE in Canada modeled after the hemophilia experience in Canada [6]. CHAES/SAHC organized an international HAE consensus meeting held in Toronto, Ontario, Canada October 2003 and from this came the first Canadian International Consensus for the diagnosis, therapy, and management of HAE [7]. The 2003 Toronto HAE Consensus meeting was held under the sponsorship of the Canadian Hematology Society and was the first meeting of the Network of Rare Blood Disorder Organizations (NRBDO; http://www.hemophilia.ca/en/about-the-chs/collaboration/network-of-rare-blood-disorder-organizations/) for Canada, and was updated in 2007 [1] through the NRBDO, the 6^th ^International C1-INH Deficiency Workshop and the 2010 Canadian HAE Consensus Conference being held in Toronto, Ontario, Canada May 15^th ^and 16^th^, 2010 sponsored by CHAEN/RCAH, the Canadian Society of Allergy and Clinical Immunology, the University of Calgary, and funded by an unrestricted educational grant from CSL Behring http://www.haecanada.com/m.php?p=ehome. The updated Canadian Consensus will be submitted to the Journal: *Allergy Asthma and Clinical Immunology*, the official Journal of the CSACI/SCAIC [8]; http://www.aacijournal.com/.

### Canadian Hereditary Angioedema Network (CHAEN)/Réseau Canadien d'Angioédème Héréditaire (RCAH)

CHAEN/RCAH is an informal organization modeled after the Association of Hemophilia Clinic Directors of Canada http://www.ahcdc.ca/. Currently, the Chair is Dr. Tom Bowen, University of Calgary http://tbowen@pol.net and the Vice-Chair Western Canada is Dr. Bruce Ritchie, University of Alberta and the Vice-Chair Eastern Canada is Dr. Jacques Hebert, Laval University. The CHAEN/RCAH website is: http://www.haecanada.com/ and the CHAEN/RCAH Webmaster is John Brosz. CHAEN/RCAH Clinic Directors collaborate with the HAE International Medical Advisory Panel http://www.haei.org/?q=node/290 and attempt to harmonize with their global initiatives including data registry and promotion of research. Our CHAEN/RCAH Clinic Directors Group and Patient Advisory Committee will work to prevent duplication of activities, minimize overlap of efforts, and work towards exchange of data base registry data in an unlinked fashion with international groups and push for open publication of treatment protocols. CHAEN/RCAH tries to meet every two years (first meeting was in conjunction with the Canadian Society of Allergy and Clinical immunology, Edmonton, September 2007). We try to update the Canadian HAE Consensus protocols, involve the CHAEN/RCAH Patient Advisory Group, involve the CHAEN/RCAH Clinic medical and paramedical staff, and foster research in HAE management in Canada. People wishing to be involved in various aspects of CHAEN/RCAH should contact Dr. Tom Bowen, http://tbowen@pol.net.

### Canadian HAE comprehensive care clinics

CHAEN/RCAH has collaborated with the Network of Rare Blood Disorder Organizations (NRBDO; http://www.hemophilia.ca/en/about-the-chs/collaboration/network-of-rare-blood-disorder-organizations/) to propose comprehensive care clinics across Canada for the treatment of various rare blood disorders including HAE. CHAEN/RCAH and the proposed comprehensive cares clinics for Canadian HAE care and home therapy are modeled after the Canadian Hemophilia Clinic Directors Group model http://www.ahcdc.ca/. The proposal for comprehensive care clinic makeup for Canadian HAE clinics is enclosed in Appendix 1. Home therapy/self-treatment has been the standard in Hemophilia Care since Dr. Hanna Strawczynski and the group at Montreal Children's Hospital published their experience in 1973 [9].

### HAE diagnosis

**Figure 1:**
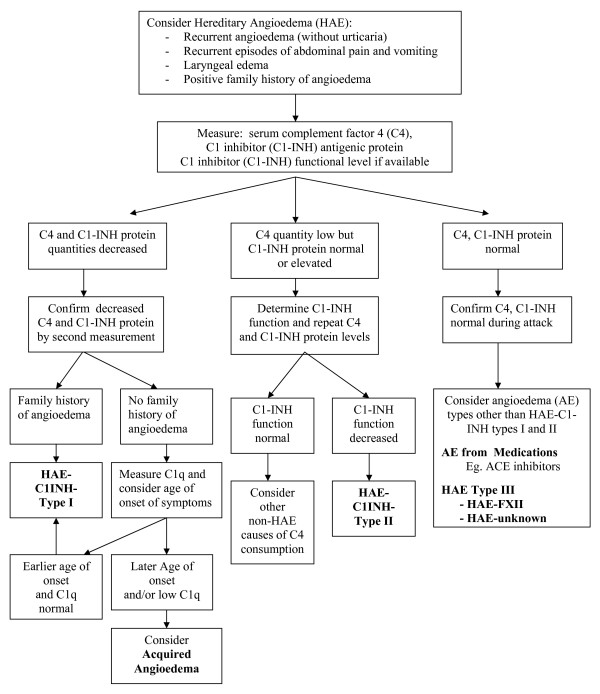
**Hereditary Angioedema - HAE - Diagnostic Algorithm**.

Diagnosis of HAE should be confirmed in laboratory centres regularly performing investigations into angioedema patients and able to perform C1-INH functional levels http://www.haecanada.com/files/DiagnosticLabsContacts.dochttp://www.haecanada.com/files/DiagnosticLabTable.doc. The diagnostic approach to angioedema without urticaria was reviewed by Zingale et al [4] and the diagnostic algorithm approach has been reviewed in previous Canadian Consensus documents and updated in the latest Canadian 2010 HAE Consensus http://www.haecanada.com/files/DiagnosticAlgorithm100527.pdf; (see Figure 1); [8]. Careful sample handling is essential to ensure accurate C1-INH functional results http://www.haecanada.com/files/DiagnosticSampleHandling.doc.

HAE patients with confirmed diagnosis are encouraged to carry wallet cards outlining their diagnosis and usual therapy http://www.haecanada.com/m.php?p=edownloads.

### HAE management

#### Prophylaxis

**Figure 2:**
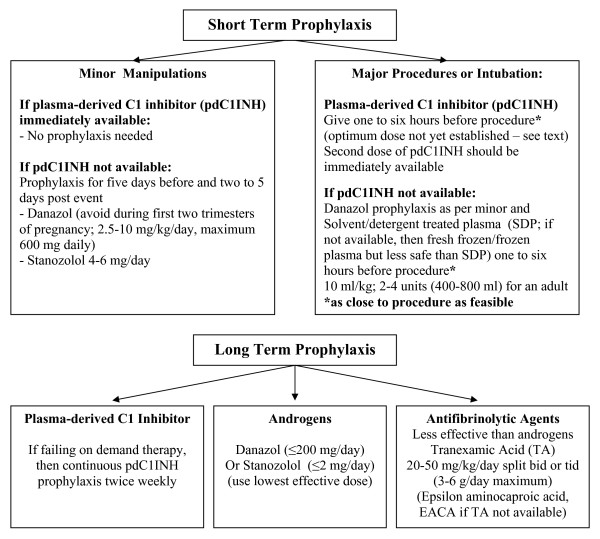
**Hereditary Angioedema - HAE - Prophylaxis Algorithm**.

In Canada, prophylaxis of angioedema events follows the Canadian and International Consensus Guidelines using tranexamic acid, anabolic steroids or C1 inhibitor replacement therapy (C1INHRP) on demand http://www.haecanada.com/files/ProphylaxisChart100527.pdf; (see Figure 2); [8].

#### Therapy

**Figure 3:**
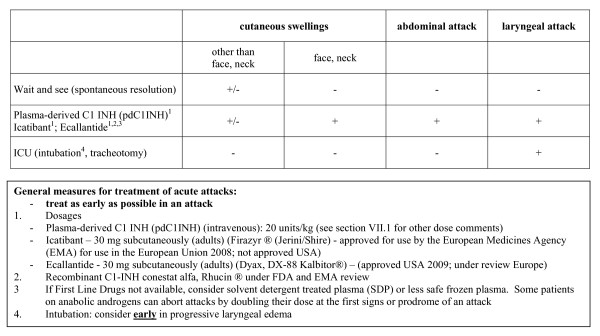
**Treatment of Acute Hereditary Angioedema - HAE - Attacks**.

In Canada, blood products are provided without charge to the patient and are funded through an interprovincial-territorial funded government program. There is a central distribution system through Canadian Blood Services for nine of the ten provinces and three Territories or through Hema-Quebec in Quebec so national statistics are available for fractionation blood products. Non-blood products used in HAE are harder to track depending on how the product is distributed. Intravenous C1 inhibitor replacement therapy (C1INHRP, Berinert^®^, CSL Behring) has been licensed for use in Canada in 2010. Icatibant (Firazyr^®^, Shire Pharmaceuticals), ecallantide (Kalbitor^®^, Dyax), and recombinant C1-INH (Rhucin^®^, Pharming Group NV) have been on clinical trial in Canada. C1INHRP has been licensed in the United States (Cinryze^®^, ViroPharma Incorporated, licensed for prophylaxis of angioedema events; Berinert^®^, CSL Behring, licensed for therapy of angioedema events). Ecallantide (Kalbitor^®^, Dyax) has been licensed in the United States for therapy of angioedema events. Icatibant (Firazyr^®^, Shire Pharmaceuticals) has been licensed in Europe for therapy of angioedema events. Recombinant C1-INH (Rhucin^®^, Pharming Group NV) is under investigation for therapy of angioedema events on both sides of the Atlantic. We support the treatment algorithm proposed in the 2010 International Consensus Algorithm for the Diagnosis, Therapy and Management of Hereditary Angioedema http://www.haecanada.com/files/TreatmentChart100527.pdf;(see Figure 3);[8].

**Figure 4:**
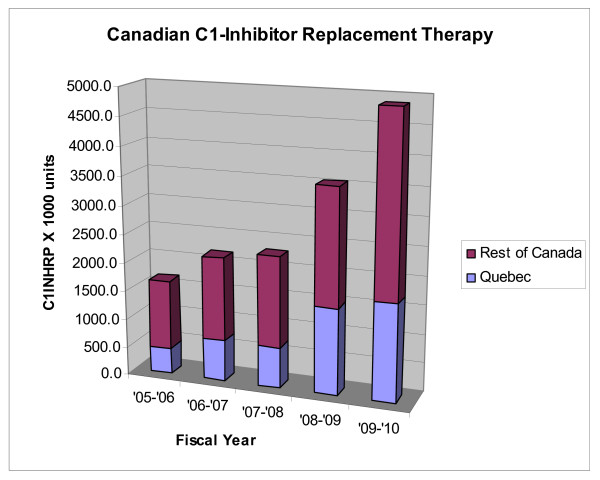
**Canadian C1-Inhibitor Replacement (C1INHRP) Therapy**.

In Canada, the use of C1INHRP has been escalating and we currently transfuse 4.87 millions units of C1INHRP per annum for the fiscal year 2009/2010 (see Figure 4) (data supplied by Dr. Francine Decary from Hema-Quebec and Keith Buchanan from Canadian Blood Services). For the fiscal year 2009/2010, 1,712,500 units were infused in Quebec (population 7,828,900; 219,000 units per million population per annum); 3,160,000 units were infused in the rest of Canada (population 25,911,000; 122,000 units per million population per annum); 144,000 units infused per million population per annum for Canada total. Like other reports, we estimate an incidence of 1:10,000 to 1:50,000 HAE patients - 20 to 100 HAE patients per million population. Statistics Canada estimates the 2010 Canadian population at approximately 33,739,900 which would predict 675 to 3374 HAE patients in Canada. If one assumes an average infusion of 1000 units C1INHRP (two vials of 500 units each vial), this translates to a current utilization of 1.4 to 7.2 infusions of C1INHRP per patient per year (includes infusions in acquired C1INH deficiency meaning these are likely high estimates per HAE patient). This has increased from our report in 2003 where the utilization rate was between 0.3 and 1.7 infusions per HAE patient per year [6]. Without an active national patient registry and without an active replacement product tracking system, these data remain guesses and are not accurate. We propose instituting a national data base registry for HAE patients and a national tracking system for replacement product utilization for HAE in Canada again modeled after the Hemophilia Care Program in Canada. Dr. Bruce Ritchie is undertaking rewriting of the Hemophilia Program blood product tracking program (Canadian Hemophilia Assessment and Resource Management System, CHARMS) and will hopefully include C1INHRP and other HAE treatment product tracking. This utilization is approaching the previously reported figures from Drs. Cicardi and Zingale from their clinic in Milan where their patients received an average of 3.85 infusions per year for laryngeal edema, 7.93 infusions per year for abdominal edema, and 1.57 infusions per year for cutaneous edema [10]. We speculate that the increased utilization is due to improved diagnosis, increased patient awareness of prophylaxis and treatment options, and increased physician awareness of the diagnosis and management of this disorder. We hope that the several national and international conferences hosted in Canada in conjunction with the Canadian Hematology Society and the Canadian Society of Allergy and Clinical Immunology have brought this about raising the standard of care closer to that of many European Countries and showing utilization figures now approaching those of European countries with mature HAE management programs in place and where Berinert^® ^has been available for therapy for about 25 years.

### Home therapy

Again modeling the Hemophilia Home Care program, some HAE patients receive home care self or assisted administration on demand. Example of self or assisted administration may be found on the CHAEN/RCAH website: http://haecanada.com/infusion/ (see Appendix 1). Other home therapy information and standards of care are reviewed by Dr. Hilary Longhurst [11,12]. In centres where home therapy has not yet been instituted, implementation of such HAE home therapy can be most rapidly accomplished by partnering with the local Hemophilia Home Care Clinic. We hope Comprehensive Care Clinics for HAE and other rare blood disorders will become established across Canada in parallel and partnership with Hemophilia clinics and Rare Blood Disorder clinics across Canada. Patients with rare disorders such as blood disorders receive optimum care through such Comprehensive Care Clinics and teams specialized in management of such complex disorders.

Some CHAEN/RCAH Physicians and clinics interested in HAE are listed on the CHAEN/RCAH website: http://www.haecanada.com/files/ChaenClinics.doc.

### Summary

We believe management of HAE in Canada has improved over the past decade thanks to the efforts of first the Canadian Hereditary Angioedema Society (CHAES)/Société d'angioédème héréditaire du Canada (SAHC) and then the Canadian Hereditary Angioedema Network (CHAEN)/Réseau Canadien d'Angioédème Héréditaire (RCAH) http://www.haecanada.com. CHAEN/RCAH has a patient advisory group that has evolved from the original CHAES/SAHC and it is hoped that the HAE Patient Group will again formally organize and replace the current patient advisory committee of CHAEN/RCAH (this is being worked on by Barbara Mako, current Patient Advisory Group Chair). Clinical research in diagnosis, therapy, and management continues in HAE clinics in Canada and it hoped more clinics will join CHAEN/RCAH and become involved in such clinical research. Comprehensive Care Clinics for HAE are slowly developing and we hope these will continue to evolve and collaborate with the National Rare Blood Disorders Organization (NRBDO) and the NRBDO clinics evolving there. Until new therapeutic products become licensed in Canada, use of these is under clinical trial studies or available through Health Canada Special Access Program. We are excited that the first licensing of a therapeutic product has finally occurred in 2010 (Berinert^®^) and anxiously wait licensing of other therapeutic options for HAE patients. CHAEN/RCAH members will remain involved in the ongoing development of international consensus approach and evidence based guidelines for HAE management.

We should remember: "It can be done - It must be done for the sake of our patients" (Tom Bowen).

## Competing interests

The authors declare that they have no competing interests affecting preparation of this manuscript. TB, BR, and JH have been involved in clinical research or educational events involving CSL Behring, Dyax, Jerini, Pharming, ViroPharma, Shire.

## Authors' contributions

TB prepared the manuscript. JB, KB, JH, and BR read, revised and approved the final manuscript.

## Appendix 1

Comprehensive Care Clinics for Hereditary Angioedema - 2010 05 27

**(Modified by permission from: **http://www.haecanada.com - comprehensive care clinics)

Comprehensive Patient Care Clinics: Clinical care, Education, and Research

Comprehensive care for HAE is based on the recognition that HAE is a chronic disease and care is complex, requiring a highly specialized and multidisciplinary approach. A comprehensive care clinic must provide accountability for in-hospital and home use of expensive and potentially toxic treatments, track outcomes (both beneficial and adverse), and develop and meet Standards of Care for HAE.

Comprehensive HAE Clinics will Provide:

1 Best Clinical Treatment outcomes including:

a. a comprehensive care team made up of nurse coordinator, clinician, social worker, data manager, pain management specialist, genetic counselor, and administrative support;

b. access to specialized diagnostic testing;

c. access to home treatment;

d. a networked Patient Information System to facilitate product recalls - collect data on therapy outcome measures and safety, and facilitate participation in clinical trials

e. access to clinical advances as they become available;

f. access to 24 hour support;

g. access to up-to-date standards of care, including standardized wallet cards;

h. tracking and intermittent audit of quality outcomes including beneficial and adverse outcomes through secure, comprehensive and networked data management.

2 Education of patients and staff regarding:

a. responsible Self/Family Care (home care model) with home and self infusion/administration instruction and support;

b. developments in the cause, diagnosis, treatment, outcomes, and prognosis of HAE

c. changes in the administrative management of the clinic

3 An environment conducive to research including:

a. access to and support for clinical trials of new treatments;

b. access to and support for translational research in diagnosis and prognosis;

c. access to and support for psychosocial research such as quality of life studies.

4 An advisory or oversight board with patient group representation for each clinic
